# Median Nerve Repair with Autologous Sciatic Nerve Graft: A Case Report

**DOI:** 10.5402/2011/120367

**Published:** 2011-04-26

**Authors:** Brian T. Ragel, Gregory C. Park, Sid Brevard

**Affiliations:** ^1^Department of Neurological Surgery, Oregon Health & Science University, 3303 SW Bond Avenue, Portland, OR 97239, USA; ^2^Department of Reconstructive Surgery, Mike O'Callaghan Federal Hospital, Nellis Air Force Base, NV 89191, USA; ^3^Division of Trauma and Surgical Critical Care, University of South Alabama College of Medicine, Mobile, AL 36688-0002, USA

## Abstract

*Background*. Peripheral nerve injury treatment options are limited to primary nerve repair, nerve grafting, and tendon transfers. In this case, a large suitable donor site was easily accessible and delayed grafting was indicative of poor prognosis. *Case Description*. A 25-year-old soldier presented to a military hospital in Afghanistan following a roadside bomb attack. The patient had a medial shrapnel wound in the bicipital groove with a cool pulseless hand and catastrophic lower extremity injuries. Bilateral above-the-knee amputations (AKAs) and exploration of the medial shrapnel wound were undertaken. A 7 cm traumatic defect in the median nerve was repaired with interpositional sciatic nerve graft harvested from the AKA. *Conclusion*. Recovery of motor function after nerve grafting is dependent on motor axons reinnervating target muscles, making proximal nerve injuries problematic. We identify a potential nerve harvest site in patients with lower extremity amputations in need of long segment nerve repairs.

## 1. Introduction

Options for treating peripheral nerve injuries include primary nerve repair, nerve grafting, and tendon transfers [1–3]. Recovery of motor function after nerve grafting is dependent on motor axons reinnervating target muscles before neuromuscular junction degeneration [1–3]. This makes proximal nerve injuries or long nerve gaps problematic because of the time it takes for regenerating axons to reach target muscles before they become reinnervation resistant, with any delays in treatment further worsening outcomes. Autologous nerve grafts (e.g. sural nerve) are preferred but are limited by graft availability, donor site morbidity, and intolerable neuropathic pain [1–3].

## 2. Case Report

A 25-year-old soldier presented to a military hospital in Afghanistan following a roadside bomb attack. The patient had multiple shrapnel wounds to the chest and upper and lower extremities. Examination of the right upper arm revealed a medial shrapnel wound in the bicipital groove with a cool pulseless hand. In addition, the patient had catastrophic lower extremity injuries that required bilateral above-the-knee amputations. 

**Figure 1:**
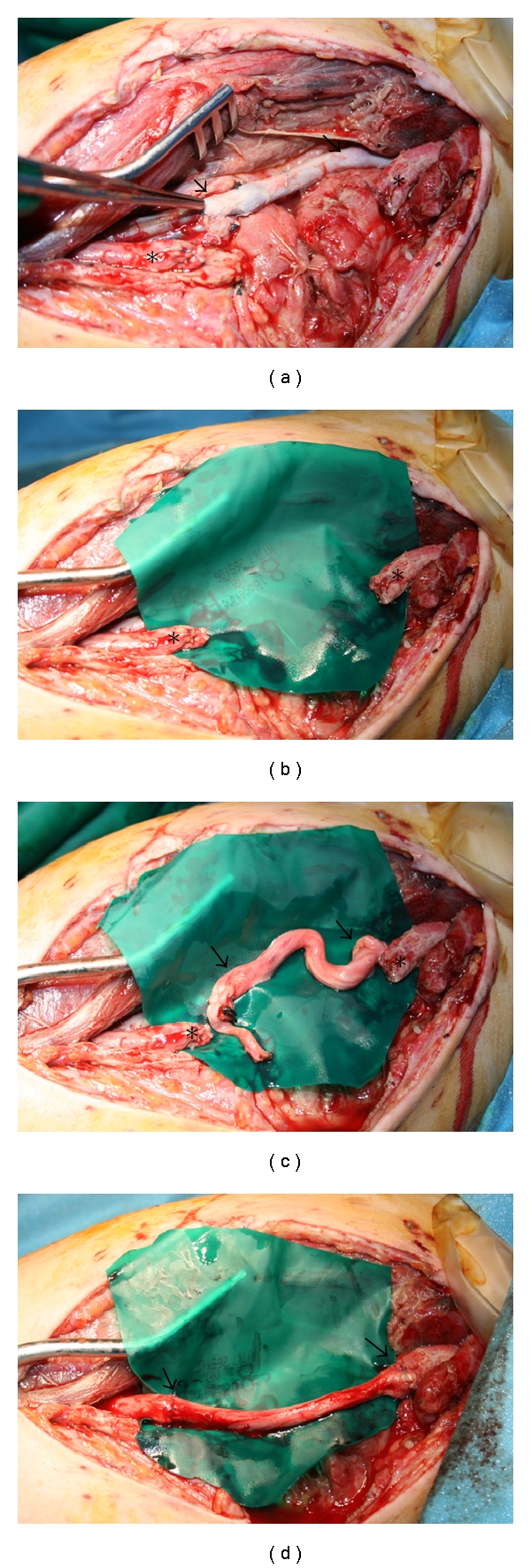
(a–d) Intraoperative photographs showing the medial aspect of right upper extremity exposure with a retractor placed in the bicipital groove. (a) Photograph showing brachial artery repair with interpositional saphenous vein graft (arrows). (b) Isolated median nerve stumps with 7 cm defect (asterisks). (c-d) Sciatic nerve harvested and interposed between median nerve stumps using 6-0 Prolene suture.

The patient underwent bilateral above-the-knee amputations (AKAs) and exploration of the right upper extremity shrapnel wound. Wound exploration revealed traumatic defects in both the brachial artery and the median nerve (Figure 1(a)). The brachial artery was repaired with saphenous vein harvested from the left leg stump (Figure 1(a)). The median nerve injury (Figure 1(b)) was repaired with interpositional sciatic nerve graft (Figure 1(c)). The sciatic nerve was identified alongside the femoral artery in the popliteal fossa of the left AKA, and an 8 cm length was harvested. After median nerve stumps were trimmed, the sciatic nerve ends were trimmed and anastomosed to both median nerve ends with 6-0 Prolene sutures (Figure 1(d)).

## 3. Discussion and Comments

Options for treating peripheral nerve injuries include primary nerve repair, nerve grafting, and nerve transfers [1–3]. Primary nerve repair is possible when nerve ends can be anastomosed in a tension-free fashion, usually in an end-to-end fashion. Nerve grafts are necessary in order to bridge physical or functional gaps in nerves [4]. Autologous nerve grafts are preferred with donor sites to include sural, superficial radial, and medial cutaneous sensory nerves [4]. Autologous grafts are limited by graft availability, donor site morbidity, and intolerable neuropathic pain [1–3]. Transfer of neighbouring motor fascicles to injured nerves can be accomplished in brachial plexus injuries with donor sites to include the dorsal scapular, accessory, ulnar, and intercostal nerves [4]. Ultimately, recovery of motor function after nerve grafting is dependent on motor axons reinnervating target muscles before neuromuscular junction degeneration [1–3]. This makes proximal nerve injuries or long nerve gaps problematic because of the time it takes for regenerating axons to reach target muscles before they become reinnervation resistant, with any delays in treatment further worsening outcomes. 

In this case, we elected for early repair because a large suitable donor site was easily accessible and the overall poor prognosis of delayed grafting [1, 2]. Unfortunately, no follow-up information is available on this patient so we are unable to confirm surgical outcome; however, we identify a potential nerve harvest site in patients with lower extremity amputations in need of long segment nerve repairs.
